# Cardiovascular-Kidney-Metabolic Syndrome: Development of an ICD-10-CM Coding Framework

**DOI:** 10.2196/91827

**Published:** 2026-06-01

**Authors:** Minzhe Zhao, Junhao Zhang, Huiyun Wang, Chongjun Xu, Jiahui Li, Chenghua Li, Xuemei He, Xueying Zheng

**Affiliations:** 1 Department of Endocrinology and Metabolism, The First Affiliated Hospital of USTC, Division of Life Sciences and Medicine, University of Science and Technology of China, 17 Lujiang Road, Luyang District, Hefei, Anhui, 230001, China, +86 13750400548; 2 Graduate School, Bengbu Medical College, Bengbu, Anhui, China; 3 Pan-Vascular Management Center, The First Affiliated Hospital of USTC, Division of Life Sciences and Medicine, University of Science and Technology of China, Hefei, Anhui, China; 4 Outpatient Department, The First Affiliated Hospital of USTC, Division of Life Sciences and Medicine, University of Science and Technology of China, Hefei, Anhui, China

**Keywords:** cardiovascular-kidney-metabolic syndrome, CKM syndrome, ICD-10-CM, coding, risk stratification, chronic kidney disease, cardiovascular disease, metabolic syndrome, diabetes mellitus, hypertension

## Abstract

**Background:**

Cardiovascular-kidney-metabolic (CKM) syndrome is a multisystem construct describing the intertwined progression of cardiometabolic risk factors, chronic kidney disease (CKD), and cardiovascular disease. The American Heart Association (AHA) proposed CKM stages (0‐4) for risk stratification and prevention. However, CKM lacks a single *ICD-10-CM* (*International Classification of Diseases, Tenth Revision, Clinical Modification*) code, which hinders standardized stage identification in electronic health records and claims data.

**Objective:**

This study aimed to develop an AHA-aligned *ICD-10-CM* coding framework as an implementation template that operationalizes CKM stages 0‐4 for reproducible cohort identification and stage-based analyses in real-world data.

**Methods:**

We mapped AHA CKM stages (0‐4) to *ICD-10-CM* diagnosis code sets using Fiscal Year 2026 conventions, code-set engineering best practices, and clinician review. To improve reproducibility, we defined a hierarchical staging algorithm, co-occurrence rules, and recommended lookback and encounter-confirmation thresholds. Stage 3 includes guidance for electronic health record–enhanced ascertainment and claims-only proxies.

**Results:**

We provide stage-specific *ICD-10-CM* code sets for CKM stages 0‐4. Stage 1 captures excess or dysfunctional adiposity or prediabetes. Stage 2 captures established metabolic disease and earlier-stage CKD. Stage 3 captures subclinical cardiovascular injury or very high-risk CKD. Stage 4 captures overt clinical cardiovascular disease events, with or without kidney failure.

**Conclusions:**

This implementation framework enables transparent, reproducible CKM staging in real-world datasets and supports stage-based epidemiologic and health-system applications. Empirical validation and local implementation testing are needed before clinical deployment.

## Introduction

### The Cardiovascular-Kidney-Metabolic Syndrome: An Integrative Health Construct

Cardiovascular-kidney-metabolic (CKM) health describes bidirectional interactions among metabolic risk factors, chronic kidney disease (CKD), and cardiovascular disease (CVD) that can mutually amplify disease progression [1,2]. CKM health is not a separate syndrome; it reframes the common clustering of obesity, type 2 diabetes mellitus (T2DM), and hypertension that accelerates both renal and cardiac decline [3-5]. CKM often begins with excess or dysfunctional adiposity and is characterized by the clustering of dysglycemia, hypertension, dyslipidemia, and systemic inflammation. These processes jointly accelerate damage to the heart, kidney, and vasculature and increase the risk of adverse cardio-renal events [6]. The American Heart Association (AHA) has therefore emphasized a shift from siloed management to stage-based prevention across the CKM continuum.

CKM syndrome reflects a reinforcing cycle—excess or dysfunctional adiposity, insulin resistance, and chronic inflammation. These factors create hemodynamic and metabolic stress on the kidneys and vasculature. Adipose tissue–derived inflammatory mediators and oxidative stress promote hypertension, dyslipidemia, and T2DM, which in turn accelerate CKD progression and atherosclerotic and heart failure (HF) phenotypes. This integrated construct motivates stage-based prevention and treatment strategies that address upstream drivers and downstream target-organ injury rather than treating each condition in isolation [3].

### The AHA Staging Framework for CKM (Stages 0-4)

The AHA proposed a 5-stage CKM framework (stages 0‐4) that links upstream cardiometabolic risk to downstream subclinical and clinical cardiovascular and kidney disease [1,2]. These stage definitions provide a shared language for risk stratification and support stage-appropriate prevention and management across the CKM continuum [3].

The AHA CKM staging scheme is defined as follows [1,2]:

- Stage 0: No CKM risk factors. Individuals with normal BMI and waist circumference, normoglycemia, normotension, a normal lipid profile, and no evidence of CKD or subclinical or clinical CVD.
- Stage 1: Excess or dysfunctional adiposity. Individuals with overweight or obesity, abdominal obesity, or dysfunctional adipose tissue, without the presence of other metabolic risk factors or CKD. This includes BMI≥25 kg/m² (or ≥23 kg/m² if Asian ancestry), waist circumference ≥88 cm in women or ≥102 cm in men (or ≥80 cm in women and ≥90 cm in men if Asian ancestry), or dysfunctional adiposity defined as hyperglycemia or prediabetes (fasting blood glucose 100‐124 mg/dL or hemoglobin A1c 5.7%‐6.4%).
- Stage 2: Metabolic risk factors and CKD. Individuals with metabolic risk factors (hypertriglyceridemia, hypertension, metabolic syndrome, and diabetes), or moderate- to high-risk CKD (per Kidney Disease: Improving Global Outcomes [KDIGO] eGFR [estimated glomerular filtration rate]-albuminuria risk categories).
- Stage 3: Subclinical CVD in CKM. Subclinical atherosclerotic cardiovascular disease (ASCVD) or subclinical HF among individuals with excess or dysfunctional adiposity, other metabolic risk factors, or CKD. Subclinical ASCVD is principally diagnosed by coronary artery calcification (subclinical atherosclerosis by coronary catheterization or computed tomography [CT] angiography also meets criteria). Subclinical HF can be diagnosed by elevated cardiac biomarkers (NT-proBNP ≥125 pg/mL; hs-troponin *t*≥14 ng/L for women and ≥22 ng/L for men; hs-troponin I≥10 ng/L for women and ≥12 ng/L for men) and/or echocardiographic parameters; a combination of biomarker and echocardiographic abnormalities indicates the highest HF risk. Risk equivalents include very high-risk CKD (G4-G5 CKD or very high risk per KDIGO) and high predicted 10-year CVD risk.
- Stage 4: Clinical CVD in CKM. Clinical CVD (coronary heart disease, HF, stroke, peripheral artery disease (PAD), and atrial fibrillation) among individuals with excess or dysfunctional adiposity, other CKM risk factors, or CKD. Stage 4 is further divided into 4A (no kidney failure) and 4B (kidney failure present).

### Rationale for an *ICD-10* or *ICD-10-CM* Coding Approach

A key barrier to implementing CKM in practice is the absence of a single *ICD-10* (*International Classification of Diseases, Tenth Revision*) code that represents the entire syndrome. This limits standardized CKM identification in electronic health record (EHR) and claims-based systems used for clinical care, population health management, and epidemiologic research [7-10]. Without a CKM-specific *ICD-10-CM* (*International Classification of Diseases, Tenth Revision, Clinical Modification*) code, case identification is inconsistent, limiting prevalence estimation, treatment evaluation, and large-scale studies of CKM natural history and outcomes [11,12]. As a result, there is a need for developing clear, reproducible, and AHA-compliant operational definitions to create mapping between *ICD-10-CM* codes and CKM’s various pathological stages. This will support more consistent identification of CKM-related phenotypes, more transparent stage assignment, and more reproducible cohort construction and outcome ascertainment across health care systems and research datasets [1,2].

There are several reasons for establishing a uniform *ICD-10-CM* approach to operationalize CKM stages. First, standardized coding enables automated identification of patients who meet stage 1‐2 criteria (eg, excess or dysfunctional adiposity, prediabetes, or hypertension) for screening, prevention programs, and longitudinal monitoring in routine care settings [13]. Second, consistent code sets support reproducible cohort construction and outcome ascertainment across health systems, improving comparability of observational studies and enabling pragmatic evaluation of prevention and treatment strategies in real-world data environments [14-16]. Finally, a shared staging framework facilitates communication across multidisciplinary teams and aligns documentation, analytics, and care pathways around the AHA CKM construct [15].

### Study Objectives

The objectives of this manuscript are to (1) present an AHA-aligned *ICD-10-CM* code set mapping CKM stages 0‐4 to existing diagnosis codes, (2) specify operational rules and assumptions for applying the algorithm in claims and EHR data, and (3) summarize implementation considerations and limitations to support reproducible use and future validation.

## Methods

### Code Set Development and Sources

We aligned CKM stage definitions to the 2023 AHA CKM construct and translated stage-defining phenotypes and outcomes into *ICD-10-CM* diagnosis code sets. Code candidates were assembled from the *ICD-10-CM* Tabular List and Index and refined using code-set engineering guidance and clinical domain knowledge. The framework is explicitly scoped to US *ICD-10-CM* (not ICD-10-CN or other national modifications). We used Fiscal Year 2026 (FY2026) *ICD-10-CM* conventions (effective October 1, 2025) to reflect current code structures and recent additions (eg, adult obesity class codes and T2DM in remission). For implementation and reproducibility, the full stage-specific code sets and logic specifications are provided in machine-actionable appendix files, with JSON as the authoritative specification and CSV as a flattened companion file for review and audit (Multimedia Appendix 1).

### Algorithm Rules and Operational Assumptions

To improve reproducibility, we specify a hierarchical staging rule in which the highest qualifying stage is assigned (stage 4 supersedes 3, which supersedes 2, etc). For claims-based implementations, chronic conditions (eg, obesity, hypertension, and CKD) can be defined using ≥2 outpatient claims on different dates (eg, ≥30 d apart) or ≥1 inpatient claim within a prespecified lookback window (eg, 1‐2 y), whereas acute events (eg, myocardial infarction and stroke) may be defined using a single inpatient claim. Stage eligibility is determined by stage-specific co-occurrence rules aligned with AHA definitions. Stage 1 requires excess or dysfunctional adiposity without other metabolic risk factors or CKD. Stage 2 requires metabolic risk factors and/or moderate- to high-risk CKD. Stage 3 requires subclinical ASCVD or subclinical HF—or risk equivalents (very high-risk CKD [G4–G5] or high predicted 10-year CVD risk)—in individuals with adiposity, other metabolic risk factors, or CKD. Stage 4 requires clinical CVD co-occurring with adiposity, other CKM risk factors, or CKD, with optional 4A or 4B stratification by kidney failure. For claims-only implementations, KDIGO risk strata are approximated using *ICD-10-CM* proxies (primarily N18 stage codes, with R80 as an optional proxy for albuminuria or proteinuria when documented); when structured laboratory data are available, eGFR and albumin-to-creatinine ratio can be used to compute KDIGO eGFR-albuminuria risk categories directly. Because subclinical disease is incompletely captured in claims data, stage 3 should be implemented preferentially in an EHR-enhanced framework, or in EHR-only settings where structured laboratory, imaging, or echocardiographic data are available. Claims-only stage 3 should be treated as a conservative proxy, primarily reflecting very high-risk CKD and related risk-equivalent states, and should be labeled as such in analytic applications (Table 1). Stage 0 is defined clinically by normal anthropometrics and cardiometabolic profiles and is operationalized in claims as the absence of qualifying codes for stages 1‐4 within the lookback window. We followed the FY2026 *ICD-10-CM* Official Guidelines for Coding and Reporting for code selection and convention handling [17].

**Table 1.** Summary of CKM^a^ stages and corresponding *ICD-10-CM*^b^ codes.^c^^,^^d^^,^^e^

CKM stage and domain or category	Operational definition and *ICD-10-CM* code set
Stage 0	
Clinical definition (AHA^f^)	Individuals with normal BMI and waist circumference, normoglycemia, normotension, a normal lipid profile, and no evidence of CKD^g^ or subclinical or clinical CVD^h^.
Claims-based operationalization (*ICD-10-CM*)	No stage-defining diagnosis codes from stages 1‐4 are present within the lookback window.
Stage 1	
Clinical definition (AHA)	Individuals with overweight or obesity, abdominal obesity, or dysfunctional adipose tissue, without the presence of other metabolic risk factors or CKD. Criteria include BMI ≥25 kg/m^2^ (or ≥23 kg/m^2^ if Asian ancestry), waist circumference ≥88 cm in women and ≥102 cm in men (or if Asian ancestry ≥80 cm and ≥90 cm in women and men, respectively), or dysfunctional adiposity defined as hyperglycemia or prediabetes (fasting blood glucose 100‐124 mg/dL or HbA1c^i^ 5.7%‐6.4%).
Claims-based operationalization (*ICD-10-CM*)	Overweight or obesity: overweight (E66.3), obesity class 1‐3 (E66.811-E66.813), and other obesity codes (E66.8, E66.9); adult BMI codes (Z68.25-Z68.45) may be used as optional supporting documentation when available; dysfunctional adiposity proxy: prediabetes (R73.03).
Exclusion rule	Absence of stage 2‐4 core codes in the lookback window.
Supplementary (non–stage-defining) adiposity-related codes (optional)	Abnormal weight gain (R63.5); other insulin resistance codes if used locally.
Stage 2	
Clinical definition (AHA)	Individuals with metabolic risk factors (hypertriglyceridemia [≥135 mg/dL], hypertension, metabolic syndrome, and diabetes), or moderate- to high-risk CKD.
Hypertension	Essential hypertension (I10), hypertensive heart disease without heart failure (I11.9), hypertensive CKD (I12.-+ N18.-), hypertensive heart and CKD without heart failure (I13.10).
Hypertriglyceridemia	Pure hypertriglyceridemia (E78.1); expanded claims-based proxy may additionally include mixed hyperlipidemia (E78.2) to capture common mixed triglyceride-elevated phenotypes in administrative data.
Metabolic syndrome	E88.810.
Diabetes	E11.- (including uncomplicated and remission code E11.A) and other diabetes codes as appropriate (eg, E10.-, E13.-).
CKD (moderate- to high-risk; claims proxy)	CKD stage 3 (N18.30-N18.32) or CKD stage 1‐2 (N18.1-N18.2) with albuminuria and proteinuria codes (eg, R80.-) within the lookback window; exclude very high-risk CKD (N18.4-N18.5) and kidney failure (N18.6).
Supplementary risk-enhancing conditions (not stage-defining)	Nonalcoholic fatty liver disease or NASH^j^ (K76.0, K75.81); sleep apnea disorders (G47.30, G47.31, G47.33, G47.39); polycystic ovary syndrome (E28.2).
Stage 3	
Clinical definition (AHA)	Subclinical ASCVD^k^ or subclinical HF^l^ among individuals with excess or dysfunctional adiposity, other metabolic risk factors, or CKD. Subclinical ASCVD is principally diagnosed by coronary artery calcification (subclinical atherosclerosis by coronary catheterization or CT^m^ angiography also meets criteria). Subclinical HF is diagnosed by elevated cardiac biomarkers and/or echocardiographic parameters, with a combination of the 2 indicating highest HF risk. Risk equivalents include very high-risk CKD (G4-G5 CKD or very high risk per KDIGO^n^) and high predicted 10-year CVD risk.
Claims-based operationalization (*ICD-10-CM*)	Claims-based operationalization (*ICD-10-CM* core risk equivalents): very high-risk CKD (N18.4-N18.5). Claims-only proxies for subclinical ASCVD or HF are limited and may under-ascertain stage 3.
Supplementary CKD-related codes (optional)	CKD-related secondary hyperparathyroidism of renal origin (N25.81) when co-occurring with N18.4-N18.5.
Stage 4	
Clinical definition (AHA)	Clinical CVD (coronary heart disease, HF, stroke, peripheral artery disease, atrial fibrillation) among individuals with excess or dysfunctional adiposity, other CKM risk factors, or CKD; stage 4A: no kidney failure; stage 4B: kidney failure present.
Claims-based operationalization (*ICD-10-CM*)	Assign stage 4 only when a clinical CVD code co-occurs with any stage 1‐3 core code and/or CKD within the lookback window.
Coronary heart disease or angina	Chronic atherosclerotic coronary disease (I25.-), angina pectoris (I20.-).
AMI^o^	AMI (I21.-; includes I21.0–I21.3 [STEMI^p^], I21.4 [NSTEMI^q^], I21.A1 [type 2 MI^r^]), subsequent MI (I22.-).
HF	Left ventricular failure (I50.1), systolic (congestive) HF (I50.2-), diastolic HF/HFpEF (I50.3-), combined systolic & diastolic HF (I50.4-), HF unspecified (I50.9); hypertensive heart disease with HF (I11.0+ I50.-); hypertensive heart and CKD phenotype with HF (I13.0/I13.2+ I50.-+ N18.-).
AF^s^	Paroxysmal AF (I48.0), persistent AF (I48.19), chronic AF (I48.20), permanent AF (I48.21).
Peripheral artery disease	Atherosclerosis of native arteries of extremities (I70.2-), peripheral vascular disease unspecified (I73.9).
Stroke (acute events)	Nontraumatic subarachnoid hemorrhage (I60.-), nontraumatic intracerebral hemorrhage (I61.-), other and unspecified nontraumatic intracranial hemorrhage (I62.-), cerebral infarction/ischemic stroke (I63.-), stroke not specified as hemorrhage or infarction (I64), sequelae of cerebrovascular disease (I69.-), personal history of TIA^t^ and cerebral infarction without residual deficits (Z86.73).
Claims-based operationalization (*ICD-10-CM*)	End-stage renal disease (N18.6), dependence on renal dialysis (Z99.2). Kidney transplant status (Z94.0+ N18.-).
Kidney failure (stage 4b)	ESRD^u^ (N18.6) and/or dialysis dependence (Z99.2), with optional transplant status codes when relevant.

a CKM: cardiovascular-kidney-metabolic.

b *ICD-10-CM*: *International Classification of Diseases, Tenth Revision, Clinical Modification*.

c Notation: In this manuscript, a hyphen suffix denotes a code-family prefix rather than an automatically executable rule. Whether descendants are included, and whether exclusions apply, is specified explicitly in the accompanying machine-actionable appendix files (JSON/CSV). Implementers should not infer descendant inclusion from shorthand notation alone.

d The definition of metabolic syndrome (MetS) is the presence of ≥3 of the following: (1) waist circumference ≥88 cm for women and ≥102 cm for men (≥80 cm for women and ≥90 cm for men if Asian ancestry); (2) HDL-C <40 mg/dL in men or <50 mg/dL in women; (3) triglycerides ≥150 mg/dL; (4) elevated blood pressure (systolic blood pressure ≥130 mmHg or diastolic blood pressure ≥80 mmHg and/or antihypertensive medication use); (5) fasting glucose ≥100 mg/dL.

e Operational assumptions (recommended for reproducibility): assign the highest qualifying stage (4>3>2>1>0). For chronic conditions, require ≥2 outpatient claims on different dates or ≥1 inpatient claim within a defined lookback window; for acute events, a single inpatient claim may be sufficient. Implementations should prespecify timing windows and setting rules (inpatient vs outpatient) based on the data source. For Stage 3, EHR-enhanced ascertainment is preferred whenever structured laboratory, imaging, or echocardiographic data are available.

f AHA: American Heart Association.

g CKD: chronic kidney disease.

h CVD: cardiovascular disease.

i HbA_1c_: hemoglobin A_1c_.

j NASH: nonalcoholic steatohepatitis.

k ASCVD: atherosclerotic cardiovascular disease.

l HF: heart failure.

m CT: computed tomography.

n KDIGO: Kidney Disease: Improving Global Outcomes.

o AMI: acute myocardial infarction.

p STEMI: ST-segment elevation myocardial infarction.

q NSTEMI: non–ST-segment elevation myocardial infarction.

r MI: myocardial infarction.

s AF: atrial fibrillation.

t TIA: transient ischemic attack.

u ESRD: end-stage renal disease.

### Validation and Quality Assurance

We conducted internal code verification to remove non-*ICD* (*International Classification of Diseases*) labels, correct formatting, and ensure consistency with current *ICD-10-CM* conventions. We also compared the stage logic and key code families with published CKM implementation studies and relevant guideline definitions. This manuscript presents an implementation framework supported by literature review and expert verification. Empirical evaluation against chart review, registry linkage, or structured EHR reference standards remains an important next step for future work.

For claims-only implementations, we recommend defining an index date (eg, cohort entry) and applying a lookback window (eg, 12‐24 mo) to capture qualifying diagnoses. A common reproducibility pattern is to require ≥1 inpatient claim or ≥2 outpatient claims on separate dates for chronic conditions, such as hypertension, diabetes, and CKD, and then assign stage using a hierarchical rule (stage 4>stage 3>stage 2>stage 1>stage 0) so that downstream clinical events supersede upstream risk states. Sensitivity analyses can vary the lookback window and diagnosis-claim thresholds to evaluate robustness.

For EHR-enhanced implementations (preferred for stage 3), structured laboratory values and imaging results can be integrated to capture subclinical disease definitions (eg, elevated natriuretic peptides, abnormal troponin, and coronary artery calcium). When these data are unavailable, stage 3 should be treated as a conservative proxy based on available diagnosis codes, and this limitation should be explicitly reported in studies using claims-only data.

Below we illustrate a claims-based use case to demonstrate how the staging hierarchy, lookback window, and encounter-confirmation rules translate into a final CKM stage assignment.

## Results

### Overview

Table 1 presents the AHA stage definitions (0‐4) alongside claims-based *ICD-10-CM* operationalizations, with risk-enhancing conditions listed separately as supplementary (non–stage-defining) codes.

### Implementation and Use Cases

For example (claims-only), consider a 60-year-old patient with an index date of January 1, 2026, and a 24-month lookback window. Chronic conditions are confirmed by ≥2 outpatient claims on different dates (≥30 d apart) or ≥1 inpatient claim; acute events are confirmed by a single inpatient claim.

Within the lookback window, the patient has (1) two outpatient claims for essential hypertension (I10) 45 days apart, (2) two outpatient claims for T2DM without complications (E11.9) 60 days apart, (3) one outpatient claim for obesity class 3 (E66.813) with a BMI code (Z68.41), and (4) one inpatient claim for acute myocardial infarction (AMI; I21.3). No codes for kidney failure (N18.6, Z99.2) are present.

Stage assignment: The confirmed I10 or E11.9 (and obesity) qualify the patient for stage 2 (established metabolic disease). The inpatient AMI code (I21.3) is a clinical CVD event and therefore meets stage 4 criteria. Applying the hierarchical rule (4>3>2>1>0), the patient is assigned CKM stage 4A (clinical CVD without kidney failure).

For contrast, a patient with overweight or obesity (E66.3, E66.8, E66.9, and/or BMI Z68.25-Z68.29) or prediabetes (R73.03) and no stage 2‐4 core codes in the same lookback window would be assigned stage 1.

### CKM Staging and Core Metabolic Disorders: *ICD-10-CM* Coding

CKM syndrome is a multidomain construct that spans metabolic risk factors, kidney disease, and CVD [18-22]. In this section, we summarize the major *ICD-10-CM* coding domains relevant to CKM staging and risk stratification. Some conditions are stage-defining within the proposed framework, whereas others serve as risk-enhancing or supplementary features that may refine phenotyping and implementation in real-world data.

### Hypertension

Hypertension represents one of the most common and important cardiovascular risk factors in CKM syndrome [23]. As one of the key elements of CKM syndrome, hypertension is a driver of the long-term and poorly controlled coronary artery disease, myocardial infarction, stroke, and peripheral arterial disease that characterize this condition [24]. The long-term elevation of blood pressure (BP) causes an excessive workload on the heart and results in an abnormal increase in heart size (hypertrophy) and ultimately acute HF [25,26]. Also, an elevated BP is a primary factor in the development of atherosclerosis, ultimately leading to increased risk for coronary arterial disease, stroke, and peripheral arterial disease [27,28]. As a result, hypertension serves as a double-edged sword to both create CKD and worsen the control of BP due to the resulting kidney damage [29]. With the availability of the *ICD-10-CM* codes, which are detailed and hierarchical for hypertension, it is possible to document the presence of hypertension and its associated complications accurately and appropriately. Additionally, knowing the specific codes that are used in conjunction with CKM staging will be the basis for accurate clinical assessments and appropriate management of all patients with CKM syndrome [1].

In *ICD-10-CM*, hypertensive heart disease is coded under I11 (I11.0 for hypertensive heart disease with HF, I11.9 without HF). When hypertension coexists with CKD, hypertensive CKD is coded under I12 with an accompanying N18 code to specify CKD stage. Combined hypertensive heart and CKD phenotypes are captured under I13 and should be paired with I50 (HF) and N18 (CKD stage) as applicable [17]. These conventions (rather than non-*ICD* labels) improve consistency when operationalizing CKM stage 2‐4 phenotypes in EHR and claims data.

### Essential Hypertension (I10)

The *ICD-10-CM* code I10 is for essential hypertension [17]. This means that a person with a diagnosis of high BP does not have any evidence of targets (such as CVD or CKD). As per CKM and CKD staging framework, a diagnosis of essential hypertension (without other metabolic risk factors or CKD) puts that person in CKM stage 2. This is due to the fact that essential hypertension is a well-established metabolic risk factor, which greatly increases the chance of developing both CVD and CKD [24,29]. Coding patients with the specific I10 code can help identify these individuals as early CKM stage 2 patients who may benefit from lifestyle modification and the use of an antihypertensive to prevent progress to later CKM stages [30]. The code I10 should only be assigned if a qualified health care provider has formally diagnosed hypertension and does not rely upon a single elevated BP measurement only [18,31]. Additionally, documentation must include evidence that the patient has persistently elevated BP that meets the criteria for a diagnosis of hypertension.

The clinical importance of identifying and coding for essential hypertension cannot be underestimated. In fact, it typically represents the first manifestation of a progressive CKM syndrome and is therefore an opportune time for health care providers to initiate treatment. It is also essential to enabling health care providers to monitor and treat patients at risk of other metabolic risk factors, such as dyslipidemia and T2DM, as well as identify early signs of target-organ injury. Early identification and appropriate intervention are critical to avoid the long-term complications associated with hypertension, which are often serious and debilitating [30]. In addition, the use of a specific code for essential hypertension will facilitate tracking the prevalence of this disease and evaluating the success of public health efforts aimed at decreasing the prevalence of hypertension in the population [32]. With the use of EHR systems, code I10 may trigger clinical decision support tools to provide reminders for BP testing, lifestyle modifications, and medication management, thus improving patient care for this major and common health problem [33,34].

### Hypertensive Heart Disease (I11)

Hypertensive heart disease is identified by *ICD-10-CM* category I11 when the medical record indicates that the patient’s chronic high BP has caused some level of structural or functional impairment to their heart [17]. Hypertensive heart disease represents a more advanced stage of disease than essential hypertension and indicates that the patient’s heart has already incurred damage due to being subjected to high BP for a prolonged period of time [35]. The most common manifestation of hypertensive heart disease is left ventricular hypertrophy (LVH), which indicates that the heart muscle has become thicker in response to increased workload. Other possible manifestations of hypertensive heart disease are HF, a condition where the heart can no longer pump sufficient amounts of blood to supply the body’s requirements [36], and would also require coding from the I50 category to specify the type of HF that is present (eg, left-sided HF [I50.1] or HF with preserved ejection fraction [I50.3]) [37]. Hypertensive heart disease should be staged according to clinical phenotype rather than diagnosis label alone. Hypertension without evidence of cardiac structural or functional involvement is consistent with CKM stage 2. Hypertensive heart disease without HF, when it reflects subclinical cardiac remodeling or dysfunction, is more appropriately aligned with CKM stage 3. Hypertensive heart disease with HF should be classified as CKM stage 4 [1,2].

The I11 code category is essential in accurately capturing the patient’s level of clinical severity and in determining how to best manage their care. Patients with hypertensive heart disease are at a substantially increased risk for adverse cardiovascular events (such as myocardial infarction, stroke, and sudden cardiac death) compared with patients with essential hypertension alone [36]. Therefore, patients with hypertensive heart disease require more intensive monitoring and treatment, including aggressive management of BP and lifestyle modification, and often including treatment for HF or other cardiac complications [38]. To support the application of the code for hypertensive heart disease, the provision of satisfactory documentation in the patient’s medical record must connect the patient’s recorded hypertension with their associated cardiac findings. Documentation must include any diagnostic tests performed to evaluate the patient’s condition (an echocardiogram may be used to identify LVH) or clinical findings that correlate with HF [39]. By properly coding for hypertensive heart disease, health care providers can offer their patients appropriate care levels and monitor their treatment progress over time [40]. Additionally, the ability to code for hypertensive heart disease will aid researchers in identifying populations at greater risk and in developing methods to treat patients diagnosed with this formidable condition.

### Hypertensive CKD (I12)

The *ICD-10-CM* code category I12 indicates hypertension with CKD, helping distinguish hypertensive CKD from other etiologies (eg, diabetes, glomerulonephritis, and polycystic kidney disease) [17,20]. Per *ICD-10-CM* conventions, an additional N18 code should be assigned to specify the CKD stage based on clinician-documented CKD stage. In our CKM operationalization, I12 supports the presence of CKD, and the resulting CKM stage depends on CKD severity (eg, N18.1-N18.3 aligns with CKM stage 2, N18.4-N18.5 with stage 3) and whether clinical CVD codes are present (stage 4). Thus, hypertensive CKD reflects hypertensive target-organ involvement, but CKM stage assignment should follow the predefined hierarchical staging rules rather than treating I12 alone as stage 3 or 4.

As for the clinical impact of hypertensive CKD, there is a strong reciprocal relationship between hypertension and CKD. Patients with hypertensive CKD may experience accelerated progression of CKD due to lack of adequate BP control as a result of declining kidney function, and the classes of medications for the management of hypertension and CKD have overlapping functions. Hypertensive CKD reflects a bidirectional hypertension-CKD interaction that accelerates renal decline and increases risks of CVD and end-stage renal disease (ESRD) [29,41]. Management is multifaceted and typically includes BP control (often renin-angiotensin-aldosterone system inhibition) and control of coexisting cardiometabolic risk factors [42]. To support I12 assignment, documentation should explicitly link hypertension as the etiology of CKD and note the rationale when alternative causes are unlikely [43,44].

### Hypertensive Heart and CKD (I13)

*ICD-10-CM* category I13 captures combined hypertensive heart disease and CKD attributable to hypertension [17]. This phenotype indicates advanced cardio-renal target-organ injury, in which cardiac dysfunction can worsen renal perfusion and CKD can exacerbate HF via volume and electrolyte disturbances [45,46]. When using I13, documentation should specify HF type (I50) and CKD stage (N18) to preserve clinical granularity.

Patients with hypertensive heart disease and CKD have a complex medical management, because these patients typically take several medications, including multiple antihypertensives, diuretics for fluid overload, and medications for managing other cardiovascular risk factors. In addition, these patients require continuous monitoring of their fluid status, electrolyte levels, and generally, renal function. Treatment options are drastically reduced for patients with both cardiac and renal dysfunction, which leads to the need for careful risk-versus-benefit analysis for treatment options [45,47]. For instance, when considering the use of renin-angiotensin-aldosterone system inhibitors, which are of benefit to both the heart and kidney, the risk of hyperkalemia and/or further decline in kidney function may limit their usage [48]. Providers need to ensure that the medical record documents an established link between hypertension, heart disease, and CKD to support the use of the I13 code. Providers usually determine this connection through a combination of patient history, examination, and diagnostic test results. Correctly coding this complicated condition is necessary so that patients can receive the proper specialized care as well as to demonstrate to the public and for reimbursement the seriousness of their condition.

### T2DM

The CKM syndrome model has identified T2DM as the prototype metabolic disorder. Thus, T2DM represents a chronic condition caused by both insufficient insulin and insulin resistance, which leads to hyperglycemia [49]. T2DM usually occurs along with all other important aspects of CKM syndrome, especially dyslipidemia, hypertension, and increased body fat; these combinations of the various risk factors create an extremely powerful synergy and thus significantly contribute to the rapid progression of both CKD and ASCVD [1,2]. Additionally, because of the continued presence of hyperglycemia resulting from T2DM, there is a direct harm to the kidneys, resulting in the development of diabetic nephropathy and a significant number of the individuals affected by this condition will go on to develop CKD and ESRD. Moreover, T2DM further increases endothelium dysfunction, promotes inflammation, and increases oxidative stress, all of which lead to the development of atherosclerosis [50]. Therefore, T2DM will greatly influence the future development of myocardial infarction, stroke, and congestive HF seen in patients with CKM [51]. Thus, it can be concluded that the presence of T2DM without other complications would constitute an individual falling into the CKM stage 2 risk category.

*ICD-10-CM* provides a standardized diagnosis coding system for T2DM and its complications [17,52]. Using *ICD-10-CM* enables hierarchical documentation of T2DM and associated complications, providing granularity that supports operationalizing CKM staging in administrative data. Diabetes complications, such as nephropathy, are treated as advanced disease manifestations; thus, individuals with these diagnoses align with higher-risk CKM profiles. E11 is the base code category for T2DM; the fourth, fifth, and sixth characters specify the presence and type of complication, allowing more precise phenotyping. This coding granularity supports improved characterization of a patient’s overall CKM health and evaluation of interventions aimed at preventing or treating diabetic complications [53].

### T2DM Without Complications (E11.9, E11.A)

A patient diagnosed with T2DM may be assigned *ICD-10-CM* code E11.9 (“type 2 diabetes mellitus without complications”) when there is no documented long-term complication such as nephropathy, retinopathy, or neuropathy [17,52]. Individuals with uncomplicated T2DM (E11.9) plus concurrent risk factors (eg, obesity and hypertension) typically meet criteria for CKM stage 2 (established metabolic disease). CKM stage 2 represents a key prevention and intervention window for intensive risk-factor modification and cardiometabolic protection [54]. In FY2026, *ICD-10-CM* introduced E11.A (“type 2 diabetes mellitus without complications, in remission”), which aligns conceptually with consensus definitions of T2DM remission and provides a standardized way to document remission status when applicable [17,55]. Accurate coding of uncomplicated T2DM and remission status can support stratified prevention strategies and improve reproducibility in real-world CKM research [56].

### CKD

CKD is a key component of the CKM syndrome, being both a primary target organ lesion caused by metabolic dysfunction as well as a risk factor for CVD [1,2,57]. Although staging CKD is emphasized by both AHA and international guidelines using eGFR and albuminuria, follow-up appointments with nephrologists should still be performed at all stages of CKM [20]. The *ICD-10-CM* provides a set of codes for CKD that are critical for accurate risk stratification and management of patients with CKD, as well as all patients under CKM. The following codes represent the respective stages of CKD: N18.1 through N18.5 indicate CKD stages 1 through 5, while N18.6 indicates ESRD. Z94.0 denotes a patient having undergone a renal transplant [17]. Accurate recording of CKD codes is essential for following the patient’s disease course and identifying patients with CKD at high risk of cardiovascular events so that timely and appropriate medical care can be provided by nephrologists. By using the most specific coding standards available, practitioners can better capture key CKD features in the EHR (eg, CKD stage [N18], kidney failure or dialysis dependence [N18.6 and Z99.2], transplant status [Z94.0], and albuminuria or proteinuria when documented [R80]). This supports individual care and population-based CKD research [58].

### CKD Staging (N18.1-N18.5)

*ICD-10-CM* category N18 captures clinician-documented CKD stage (1-5) based on a patient’s eGFR and the degree of albuminuria [17,20]. Albuminuria should be coded separately when documented (eg, R80). Stage 1 CKD (N18.1) represents early CKD, whereas stage 5 (N18.5) indicates advanced CKD immediately preceding kidney failure (N18.6). For clinical documentation, the assigned N18 code should match the clinician-documented CKD stage at the time of the encounter. For research applications in longitudinal data, investigators should prespecify whether CKD severity is defined using the current stage, a worst-ever stage within a lookback window, or time-updated staging, and report this choice explicitly. In our CKM operationalization, CKD stages 1‐3 generally align with CKM stages 2‐3, whereas CKD stages 4‐5 indicate very high-risk CKD and typically align with CKM stage 3 (or stage 4 when clinical CVD co-occurs) [44].

### ESRD and Transplant Status (N18.6, Z99.2, and Z94.0)

*ICD-10-CM* code N18.6 refers to ESRD, the final stage of CKD requiring renal replacement therapy, such as dialysis or transplantation [17]. Dialysis dependence is captured by Z99.2, and kidney transplant status is captured by Z94.0 [59]. It should be kept in mind that even after transplantation, some patients may still have CKD because the transplanted organ may not return the patient back to baseline kidney function; therefore, the N18 code for the patient’s CKD stage should be used alongside the Z94.0. ESRD or kidney transplant status indicates a very high risk of CVD, and, as such, are generally classified as “CKM stage 4B.” The care of patients experiencing ESRD or who have had a transplant, therefore, warrants a multidisciplinary team consisting of nephrologists, cardiologists, and other specialists to ensure coordinated care between all members involved in the patient’s treatment [60]. The accurate coding for patients suffering from chronic renal failure is vital to ensure that specific specialized services are provided, and to track the outcomes of this population, which is classified as high risk.

### Obesity and Overweight

Insulin resistance, T2DM, dyslipidemia, and hypertension are strongly influenced by obesity—particularly abdominal adiposity—which is a major upstream driver of CKM progression [61,62]. To enable stage-based identification and monitoring in real-world data, *ICD-10-CM* provides diagnosis codes for overweight and obesity and companion BMI Z-codes. Importantly, adult obesity severity can be coded using the obesity class codes E66.811 (class 1), E66.812 (class 2), and E66.813 (class 3) [17]. When available, companion BMI Z-codes may be used as optional supporting documentation to improve phenotypic granularity, but they are not required when an overweight or obesity diagnosis code is already present. These codes improve phenotyping granularity compared with legacy “morbid obesity” labels and support more consistent CKM stage 1 identification.

### Adult Obesity Class 1-3 (E66.811-E66.813)

Adult obesity class codes map to BMI-based severity strata (class 1: BMI 30.0‐34.9, class 2: BMI 35.0‐39.9, and class 3: BMI≥40.0). Companion BMI Z-codes (Z68.3x-Z68.4x) may be used as optional supporting codes when available, but they are not required for stage 1 classification if an obesity diagnosis code has already been documented [17]. While legacy obesity diagnoses (eg, E66.9 and other E66 subcodes) remain in use, the class codes provide a more clinically relevant, less stigmatizing way to represent severity and can improve risk stratification and treatment planning. Within the CKM framework, higher obesity class is associated with greater risk of progression to hypertension, T2DM, CKD, and CVD, underscoring the importance of accurate and specific obesity documentation for early CKM intervention [63,64].

### Overweight and BMI Documentation (Z68)

*ICD-10-CM* codes Z68.25-Z68.29 are assigned when a person has a BMI classified as “overweight” (25.0‐29.9 kg/m²) [17,65]. Overweight is recognized as an important risk factor for the development of CKM syndrome, although it does not meet the definition of a “disease.” These codes provide the ability to consistently document the identification of overweight individuals in the EHR data and allow population-level tracking and research on the effects of obesity. When an individual meets the CKM criteria with only 1 risk factor, their condition is classified as CKM stage 1. Once another risk factor develops, such as hypertension, high blood sugar, or dyslipidemia, the severity of their CKM classification will be elevated to CKM stage 2. Therefore, BMI documentation can improve phenotypic granularity and support prevention-oriented stratification when available, but stage 1 classification does not require a BMI Z-code if an overweight or obesity diagnosis code is already documented.

### Metabolic Liver Disease

Nonalcoholic fatty liver disease (NAFLD) and its more severe inflammatory form, nonalcoholic steatohepatitis (NASH), have become well-established as part of the CKM syndrome [66]. These conditions are often referred to as “the hepatic manifestation of metabolic syndrome” and are closely linked with obesity, insulin resistance, and T2DM [67-69]. In the strict AHA-aligned staging algorithm presented here, NAFLD or NASH are treated as supplementary (risk-enhancing) conditions rather than stage-defining criteria and can be used for stratification and sensitivity analyses.

### NAFLD (K76.0)

The *ICD-10-CM* classification for “Fatty (change of) liver, not elsewhere classified” is K76.0 [17,70]. Although this classification does not specifically diagnose NAFLD, it serves to identify the existence of a fatty liver in the absence of a more defined diagnosis such as NASH. NAFLD identifies a serious level of metabolic dysfunction throughout the body and places a significant determinant of the likelihood of future events involving the heart and kidneys [71,72]. In the CKM framework, NAFLD may be diagnosed along with CKM stage 2 or higher, indicating the need for very aggressive lifestyle modification and continued follow-up that incorporates the liver, heart, and kidney in concert with one another, rather than independently.

### NASH (K75.81)

K75.81 is the code for the most advanced and inflammatory type of liver damage due to fat, known as NASH [17]. NASH causes liver inflammation and injury and can lead to scarring (fibrosis), liver failure (cirrhosis), and other health issues [73]. The presence of NASH indicates that the body has a greater amount of metabolic dysfunction than is seen with other forms of fatty liver disease and has a greater chance of developing systemic problems related to that severity of metabolic dysfunction [71,74]. When a patient is diagnosed with NASH, the likelihood that the patient will be classified as a high-risk patient on the CKM spectrum (usually CKM stage 2 or 3) and require an extensive treatment plan that manages the metabolic problems causing the NASH, as well as monitors for possible liver-related complications.

### Other Metabolism-Related Diseases and Risk-Enhancing Conditions: *ICD-10-CM* Coding

In addition to CKM syndrome’s core disorders, many other health conditions significantly increase risk (ie, “risk-enhancers”) for CKM syndrome. They are dyslipidemia, metabolic syndrome, hyperuricemia, obstructive sleep apnea (OSA), polycystic ovarian syndrome (PCOS), and CKD-related secondary hyperparathyroidism (SHPT) [67,68,75-77]. The co-occurrence of these conditions may inform CKM risk stratification, but not all of them are stage-defining. In this framework, only diagnoses explicitly mapped in the staging algorithm are used for CKM stage assignment, whereas the others function as complementary risk-enhancing conditions.

### Dyslipidemia and Lipoprotein Metabolism Disorders (E78)

Dyslipidemia is a major contributor to ASCVD and a core risk domain within the CKM syndrome [69]. In *ICD-10-CM*, most dyslipidemias and disorders of lipoprotein metabolism are captured under E78 (disorders of lipoprotein metabolism and other lipidemias), including specific codes for pure hypercholesterolemia (E78.0), pure hyperglyceridemia or hypertriglyceridemia (E78.1), mixed hyperlipidemia (E78.2), other hyperlipidemia (E78.4, including E78.41 and E78.49), and unspecified hyperlipidemia (E78.5) [17]. In the AHA CKM staging framework, hypertriglyceridemia (fasting triglycerides ≥135 mg/dL) is considered a stage 2 metabolic risk factor. Accordingly, pure hypertriglyceridemia (E78.1) is retained as the primary stage 2 code, and mixed hyperlipidemia (E78.2) may also be retained as an expanded claims-based proxy to capture mixed triglyceride-elevated phenotypes in administrative data. Broader lipid abnormalities remain generally treated as risk-enhancing rather than stage-defining conditions. Although E78.5 (hyperlipidemia, unspecified) is a billable code, more specific E78 codes should be used whenever the lipid phenotype is known, to improve clinical characterization and support more targeted lipid-lowering strategies [75,78]. The presence of dyslipidemia in conjunction with other CKM risk factors substantially increases future cardiovascular risk and underscores the need for timely risk-based intervention.

### Metabolic Syndrome (E88.810)

Metabolic syndrome represents the clustering of multiple risk factors including central obesity, hyperglycemia, hypertension, and dyslipidemia [76]. It is an important precursor state and phenotype of CKM. *ICD-10-CM* provides a dedicated code for metabolic syndrome, that is, E88.810 [17]. This code can be used when at least 3 of the classic diagnostic criteria are met, which include increased waist circumference, elevated triglycerides, reduced high-density lipoprotein cholesterol, elevated BP, and elevated fasting glucose [79]. In the AHA CKM staging framework, the presence of metabolic syndrome serves as a key marker for progression to CKM stage 2. It is also a crucial code for population screening and for the automated identification of individuals at CKM stage 2 in EHRs.

### Hyperuricemia and Gout (E79.0 and M10)

Hyperuricemia is interconnected with hypertension, CKD, insulin resistance, and obesity, and is now recognized as an independent CKM risk factor [80-82]. Urate crystals impair endothelial function, incite inflammation and oxidative stress, and accelerate atherosclerosis [83]. The *ICD-10-CM* code E79.0 is used for asymptomatic hyperuricemia, which is useful for identifying metabolically vulnerable yet oligo-symptomatic individuals. The codes M10 are used for gout, with subcategories for acute, chronic, and tophaceous presentations [17]. Hyperuricemia often accompanies CKM stages 1‐2, whereas gout—especially when coexisting with CKD or CVD—usually implies a higher CKM stage and cardio-renal risk.

### Sleep Apnea Disorders (G47.30, G47.31, G47.33, and G47.39)

OSA, characterized by repetitive upper-airway collapse during sleep, is strongly associated with hypertension, arrhythmia, HF, and insulin resistance, and is a prevalent CKM comorbidity [77]. *ICD-10-CM* code G47.33 denotes OSA (adult or pediatric). Related sleep apnea codes include G47.30 (sleep apnea, unspecified), G47.31 (primary central sleep apnea), and G47.39 (other sleep apnea) [17]. Recognition and coding of OSA facilitates early identification of high-risk individuals in CKM stages 1‐2. Continuous positive airway pressure therapy improves sleep quality and may lower BP and CVD risk, rendering OSA a modifiable risk-enhancing factor [84].

### PCOS (E28.2)

PCOS is one of the most common endocrine disorders in reproductive-age women, characterized by hyperandrogenism, anovulation, and insulin resistance, and is strongly linked to obesity, T2DM, dyslipidemia, and increased long-term CVD risk [85-87]. It is viewed as an early female-predominant CKM phenotype. The *ICD-10-CM* code E28.2 is used for polycystic ovary syndrome. Young women with PCOS frequently occupy CKM stages 1‐2 even before developing overt hypertension or diabetes [17]. Early coding and intervention (weight control, insulin-sensitizing measures, and lipid management) can delay progression to higher-risk stages.

### CKD-Related SHPT of Renal Origin (N25.81)

SHPT of renal origin is a hallmark complication of moderate-to-advanced CKD, driven by phosphate retention, impaired calcitriol synthesis, and hypocalcemia, leading to parathyroid hyperplasia and excess parathyroid hormone secretion [68]. It contributes to mineral and bone disorder, vascular calcification, LVH, and heightened CVD risk. In *ICD-10-CM*, CKD-related SHPD is specifically coded as N25.81 and is typically documented alongside N18 to indicate CKD stage (eg, N18.4+N25.81) [17]. Clinically, the emergence of N25.81 usually reflects CKM stages 3‐4 and indicates the need for integrated management of CKD-mineral bone disorder and CVD.

### CVD Coding in CKM Syndrome

CVD is the central clinical outcome of CKM syndrome and a key determinant of progression from CKM stage 3 (subclinical cardiovascular injury or very high-risk status) to CKM stage 4 (overt clinical events) [88]. In the AHA framework, stage 3 commonly corresponds to subclinical atherosclerosis and/or early cardiac dysfunction, whereas stage 4 denotes established clinical ASCVD, HF, disabling stroke, and other “hard” end-stage events [1,2]. Accordingly, systematic mapping of major cardiovascular phenotypes and their *ICD-10* or *ICD-10-CM* codes is critical for identifying CKM stage 3‐4 populations in EHRs and epidemiological databases.

### Coronary Heart Disease and Angina (I20 and I25)

Coronary heart disease encompasses a group of conditions caused by coronary atherosclerosis with luminal stenosis or occlusion, resulting in myocardial ischemia, hypoxia, and, in severe cases, necrosis [89-91]. Within the CKM paradigm, coronary disease is often the downstream “end point manifestation” after prolonged exposure to obesity, diabetes, hypertension, dyslipidemia, and CKD, and typically aligns with CKM stage 4 [57]. In *ICD-10-CM*, chronic atherosclerotic coronary disease is primarily coded under I25. Specifically, I25.10 denotes atherosclerotic heart disease of native coronary artery without angina pectoris, while native-vessel atherosclerotic disease with angina pectoris is captured under I25.11 (child codes I25.110-I25.119) [17]. Atherosclerosis of coronary artery bypass grafts or of a coronary artery of a transplanted heart with angina pectoris is captured under I25.7, whereas bypass-graft atherosclerosis of coronary artery bypass grafts without angina pectoris can be captured under I25.810. When angina is clinically documented, angina pectoris is coded under I20 (eg, I20.0 unstable angina, I20.1 angina with documented spasm, I20.2 refractory angina, I20.8 other forms, and I20.9 unspecified). Co-occurrence of I25 with E11 (diabetes), I10-I13 (hypertension), and N18 (CKD) often indicates advanced, multiorgan CKM with very high event and mortality risk, and is frequently used to define major adverse cardiovascular events in research settings.

### AMI (I21 and I22)

AMI is the most severe acute presentation of coronary disease, typically triggered by rupture of a vulnerable plaque with superimposed thrombosis, leading to complete or near-complete coronary occlusion and long-term myocardial necrosis [92]. In CKM populations, obesity, diabetes, and CKD increase plaque burden and vulnerability, markedly raising AMI risk [93]. *ICD-10-CM* classifies AMI under I21, including I21.0-I21.3 for ST-segment elevation myocardial infarction by site or wall and I21.4 for non–ST-segment elevation myocardial infarction; I21.A1 specifies myocardial infarction type 2 (supply-demand mismatch), which may occur in contexts such as severe anemia, tachyarrhythmia, or hypertensive crisis. I22 captures subsequent myocardial infarction (MI) occurring within 4 weeks (28 d) of a previous acute MI, regardless of site [17]. In CKM staging and clinical research, I21 and I22 are typically treated as “hard end points”; the presence of these codes generally indicates CKM stage 4 and warrants intensive long-term secondary prevention (eg, high-intensity lipid lowering, antiplatelet therapy, and strict BP and glycemic control) [94].

### HF (I50, I11.0, and I13)

HF represents the common final pathway of many structural and functional cardiac disorders, characterized by impaired ventricular systolic and/or diastolic function such that cardiac output fails to meet metabolic demands (or is maintained only at the expense of elevated filling pressures) [94,95]. In CKM syndrome, hypertensive remodeling, diabetic cardiomyopathy, ischemic cardiomyopathy, and CKD-related volume overload all contribute to HF development. *ICD-10-CM* codes for HF include I50.1 (left ventricular failure), I50.2 (systolic [congestive] HF), I50.3 (diastolic [congestive] HF), I50.4 (combined systolic and diastolic HF), and I50.9 (HF, unspecified). When HF is explicitly attributed to long-standing hypertension, I11.0 (hypertensive heart disease with HF) should be used, with an additional code from I50. to identify the type of HF. When hypertensive heart disease coexists with CKD and HF, I13.0 (with CKD stage 1‐4 or unspecified CKD) or I13.2 (with CKD stage 5 or ESRD) is used, with additional codes from I50 (HF type) and N18 (CKD stage). In CKM staging, the presence of I50 (or I11.0 with I50; or I13.0 or I13.2 with I50 and N18) generally places patients in CKM stage 4 (clinical HF), representing the highest cardio-renal comorbidity burden [1,2,17].

### Arrhythmias, With a Focus on Atrial Fibrillation (I48)

Atrial fibrillation (AF) is the most common long-term arrhythmia, characterized by rapid, disorganized atrial electrical activity, loss of effective atrial contraction, blood stasis, and a propensity for mural thrombus formation—making it a major cause of ischemic stroke [96]. Obesity, OSA, hypertension, T2DM, and CKD promote atrial structural and electrical remodeling, increasing AF risk [77,96]. In *ICD-10-CM*, AF and flutter are coded under I48, including I48.0 (paroxysmal AF), I48.19 (persistent AF), I48.20 (chronic AF), and I48.21 (permanent AF) [17]. Other arrhythmias common in advanced CKM include ventricular tachycardia (I47.2), premature ventricular contractions (I49.3), and ventricular fibrillation (I49.0). AF can be regarded as a CVD-equivalent condition; when combined with previous stroke (I63) or HF (I50), it typically indicates the highest-risk tier and aligns with CKM stage 4.

### PAD (I70 and I73)

PAD refers to atherosclerotic narrowing or occlusion of medium-to-large arteries outside the coronary and intracranial circulations, most commonly affecting the lower extremities [77]. It is a marker of systemic atherosclerosis and often coexists with coronary and cerebrovascular disease [97]. *ICD-10-CM* codes include I70.2 (atherosclerosis of native arteries of the extremities) and I73.9 (peripheral vascular disease, unspecified), among others [17]. In CKM staging, PAD is generally categorized as clinical ASCVD; the presence of I70 or I73 typically indicates CKM stage 4.

### Stroke (I60-I64)

Stroke, including ischemic and hemorrhagic subtypes, is among the most severe and disabling end points across the CKM spectrum. CKM individuals often exhibit clustering of hypertension, diabetes, AF, and dyslipidemia, resulting in cumulative cerebrovascular risk. In *ICD-10-CM*, acute stroke events are generally captured under I60-I64, including I60 (nontraumatic subarachnoid hemorrhage), I61 (nontraumatic intracerebral hemorrhage), I62 (other and unspecified nontraumatic intracranial hemorrhage), I63 (cerebral infarction or ischemic stroke), and I64 (stroke, not specified as hemorrhage or infarction) [17]. To improve ascertainment of previous clinical cerebrovascular disease in claims or EHR data, history or sequelae codes can also be captured, including I69 (sequelae of cerebrovascular disease) and Z86.73 (personal history of transient ischemic attack and cerebral infarction without residual deficits) [17]. The occurrence of stroke indicates CKM stage 4 and is frequently associated with substantial functional impairment and reduced quality of life, serving as a key end point for evaluating integrated cardio-renal-metabolic prevention.

## Discussion

### Principal Findings

In this study, we developed an AHA-aligned *ICD-10-CM* coding framework to operationalize CKM syndrome stages 0‐4 in EHRs and claims data. The framework provides stage-specific code sets, a hierarchical staging algorithm, and practical rules for co-occurrence, lookback periods, and encounter confirmation. These components provide a transparent and reproducible approach for CKM staging in real-world datasets.

### Clinical and Research Implications of a Standardized Coding Framework

Establishing a consistent *ICD-10-CM* coding framework for CKM has important clinical and research implications. Consistent coding can improve the completeness, transparency, and interpretability of patient-related data for clinicians, while supporting cross-specialty communication and the development of stage-aware decision support tools [98]. These tools will allow clinicians to automatically identify the CKM stage for their patients based on a coded diagnosis, which will trigger a clinician’s response to provide stage-specific interventions [99]. For instance, clinicians could provide lifestyle counseling to patients diagnosed with CKM stage 1 and intensive management of risk factors to patients diagnosed with CKM stage 3. On the research side, a standardized coding framework is necessary for conducting large, multicenter studies on the epidemiology, natural history, and treatment of CKM syndrome [89]. The use of a standardized code system can support more reproducible population identification, more transparent exposure and outcome ascertainment, and more consistent comparison across studies and health care systems [40,100].

### Framework Validity Scope and Comparison With Existing *ICD*-Based CKM Staging Approaches

The validity of any *ICD*-based staging framework rests on (1) content validity—whether included codes represent the stage-defining phenotypes, (2) structural validity—whether the algorithm assigns mutually exclusive stages while preserving clinical hierarchy, and (3) operational validity—whether implementation choices (eg, ascertainment windows and encounter-confirmation rules) improve reproducibility, transparency, and consistency of stage assignment across datasets.

We enhanced content validity by anchoring every stage to the AHA definitions and prioritizing granular *ICD-10-CM* codes and etiologic combination codes for kidney and cardiovascular conditions. All codes and ranges were standardized against current US *ICD-10-CM* conventions (FY2026) to avoid non-*ICD* labels and ambiguous shorthand, and we explicitly note newer codes that may be absent from older datasets (eg, E11.A; adult obesity class codes E66.811-E66.813). Structural validity is addressed through an explicit top-down algorithm that assigns the highest qualifying stage (4->0), which prevents double counting and makes precedence rules transparent.

Previously published *ICD*-based operationalizations vary and often omit key design decisions that affect classification consistency and implementation reproducibility [101]. Common limitations include incomplete capture of adiposity-only stage 1, inconsistent handling of subclinical stage 3 (often merged with stage 2 in claims data), and insufficiently specified precedence, timing, and encounter-confirmation rules [93]. In contrast, our framework (1) provides modular, stage-specific code domains; (2) separates claims-only staging from EHR-enhanced staging; and (3) operationalizes stage 3 explicitly by distinguishing an EHR-defined subclinical CVD component from a conservative claims-only definition based on very-high risk CKD. These choices improve transparency and facilitate sensitivity analyses when the available data cannot fully represent subclinical disease.

With respect to validation, the present work provides literature- and guideline-based validation (code verification and crosswalk to published CKM algorithms) rather than empirical performance metrics. We therefore recommend that future external evaluations benchmark the framework in specific target datasets using chart review, registry linkage, or structured EHR reference standards under prespecified encounter-confirmation and lookback rules [40,90,91].

### Facilitating Multidisciplinary and Coordinated Care

The CKM framework includes a focus on an integrated team-based approach to care instead of fragmented siloed care, with standardized coding using the *ICD-10-CM* for coding conditions related to CKD [102,103]. Using a shared code set across providers creates a common representation of the patient’s condition, health status, and risk profile. This supports cross-specialty communication and coordination around an integrated metabolic-renal-cardiovascular care plan. A shared code set standardizes the patient’s risk profile and supports cross-specialty coordination. For example, documenting diabetic nephropathy (E11.22) and stage 3 CKD (N18.3) can prompt proactive cardiology communication given the elevated cardiovascular risk. This coordination is central to improving outcomes in advanced CKM.

### Limitations and Challenges of *ICD-10-CM* Coding for CKM

Although *ICD-10-CM* codes enable scalable CKM case identification, several limitations should be considered. First, coding accuracy depends on the completeness and specificity of clinical documentation; underdocumentation may lead to underascertainment of CKM phenotypes and misclassification of stage [104]. Second, *ICD-10-CM* may not fully capture etiologic nuance (eg, differentiating diabetic vs hypertensive CKD when documentation is ambiguous), which can introduce heterogeneity in stage definitions across sites [105]. Third, stage 3 is inherently challenging to operationalize in claims-only data because subclinical disease is often defined by imaging findings (eg, coronary artery calcium) or biomarkers (eg, high-sensitivity troponin or natriuretic peptides) that are not reliably represented in diagnosis codes. Accordingly, stage 3 identification may require an EHR-enhanced approach (integrating structured laboratories or imaging results) or should be interpreted as a conservative proxy when using claims-only algorithms [106,107]. Finally, because *ICD-10-CM* is updated annually, code sets should be periodically reviewed and maintained to remain consistent with current conventions and guidance [108].

### Future Directions and Recommendations

Going forward, there are several basic actions that need to be taken to further develop and use a standardized *ICD-10-CM* coding framework for CKM syndrome. First, validation studies should be conducted to evaluate the coding algorithm’s performance when used in different types of health care settings and patient populations [109]. Validation studies will compare the CKM stages assigned through this automated coding framework to CKM stages assigned using traditional medical record review methods, allowing for a comparison of the accuracy and dependability between automated methods and traditional methods of determining to assign CKM stages [110]. Second, CKM staging frameworks should be incorporated into established clinical guidelines and evaluated in quality metrics. An increased awareness of the CKM syndrome among health care providers and the increased use of a standardized coding system should be an outcome of this effort. Additionally, ongoing education and training for health care providers, medical coders, and others associated with health care should be provided regarding the importance of accurate, specific, and complete CKM documentation and codification of CKM-related conditions [111]. Education and training could be provided in a variety of formats, including online training modules, workshops, and various types of educational materials. Last but not least, as CKM research progresses, the *ICD-10-CM* coding framework should be periodically reviewed for consistency with current CKM findings and be updated to include new codes as they are available [112]. The achievement of these objectives is likely to result in continued relevance and improvement of the *ICD-10-CM* coding framework for CKM syndrome. In addition, as natural language processing and information extraction mature, subclinical findings documented in unstructured EHR notes (eg, coronary artery calcium reporting, echocardiography interpretations, or biomarker results referenced in narratives) could be leveraged to improve stage 3 ascertainment when structured data are incomplete. Finally, integrating this staging framework into clinical quality measures and electronic decision support tools may facilitate scalable, stage-appropriate prevention and care pathways while enabling ongoing monitoring and iterative refinement of the code sets over time.

### Conclusion

The CKM syndrome is a growing public health concern because metabolic dysfunction, CKD, and CVD interact bidirectionally and can amplify one another over time. The AHA proposed a staged CKM framework (0‐4) to support risk stratification and stage-appropriate intervention, but the absence of a single *ICD-10-CM* code has limited standardized implementation in real-world data. In this manuscript, we provide an AHA-aligned *ICD-10-CM* operational framework that links stage-defining phenotypes and outcomes to code sets and reproducible algorithmic rules. If adopted and maintained, this framework can support consistent cohort identification, risk stratification, and outcome assessment across health systems and claims databases while enabling future empirical validation and iterative refinement.

## Supplementary material

10.2196/91827 Multimedia Appendix 1 Additional information.

## Abbreviations

AF: atrial fibrillation
AHA: American Heart Association
AMI: acute myocardial infarction
ASCVD: atherosclerotic cardiovascular disease
BP: blood pressure
CKD: chronic kidney disease
CKM: cardiovascular-kidney-metabolic
CT: computed tomography
CVD: cardiovascular disease
eGFR: estimated glomerular filtration rate
EHR: electronic health record
ESRD: end-stage renal disease
FY2026: Fiscal Year 2026
HF: heart failure
*ICD*: *International Classification of Diseases*
*ICD-10*: *International Statistical Classification of Diseases, Tenth Revision*
*ICD-10-CM*: *International Classification of Diseases, Tenth Revision, Clinical Modification*
KDIGO: Kidney Disease: Improving Global Outcomes
LVH: left ventricular hypertrophy
MI: myocardial infarction
NAFLD: nonalcoholic fatty liver disease
NASH: nonalcoholic steatohepatitis
OSA: obstructive sleep apnea
PAD: peripheral artery disease
PCOS: polycystic ovary syndrome
SHPT: secondary hyperparathyroidism
T2DM: type 2 diabetes mellitus
